# Implementation of Novel Molecular Biomarkers for Non-small Cell Lung Cancer in the Netherlands: How to Deal With Increasing Complexity

**DOI:** 10.3389/fonc.2019.01521

**Published:** 2020-01-22

**Authors:** Daan van den Broek, T. Jeroen N. Hiltermann, Bonne Biesma, Winand N. M. Dinjens, Nils A. 't Hart, John W. J. Hinrichs, Mathie P. G. Leers, Kim Monkhorst, Matthijs van Oosterhout, Volkher Scharnhorst, Ed Schuuring, Ernst-Jan M. Speel, Michel M. van den Heuvel, Ron H. N. van Schaik, Jan von der Thüsen, Stefan M. Willems, Leonie de Visser, Marjolijn J. L. Ligtenberg

**Affiliations:** ^1^Department of Laboratory Medicine, Netherlands Cancer Institute, Amsterdam, Netherlands; ^2^Department of Pulmonary Diseases, University Medical Center Groningen, University of Groningen, Groningen, Netherlands; ^3^Department of Pulmonary Diseases, Jeroen Bosch Hospital, 's-Hertogenbosch, Netherlands; ^4^Department of Pathology, Erasmus MC, University Medical Center Rotterdam, Rotterdam, Netherlands; ^5^Department of Pathology, Isala Klinieken, Zwolle, Netherlands; ^6^Symbiant Pathology Expert Centre, Alkmaar, Netherlands; ^7^Department of Pathology, University Medical Center, Utrecht, Netherlands; ^8^Department of Clinical Chemistry, Zuyderland Medical Center, Sittard-Geleen, Netherlands; ^9^Department of Pathology, Netherlands Cancer Institute, Amsterdam, Netherlands; ^10^DNA Pathology, Department of Pathology, St. Antonius Ziekenhuis, Nieuwegein, Netherlands; ^11^Clinical Laboratory, Catharina Hospital, Eindhoven, Netherlands; ^12^Department of Pathology, University Medical Center Groningen, University of Groningen, Groningen, Netherlands; ^13^Department of Pathology, GROW-School for Oncology and Developmental Biology, Maastricht University Medical Center, Maastricht, Netherlands; ^14^Department of Pulmonary Diseases, Radboud University Medical Center, Nijmegen, Netherlands; ^15^Department of Clinical Chemistry, Erasmus MC, University Medical Center Rotterdam, Rotterdam, Netherlands; ^16^Roche Diagnostics, Almere, Netherlands; ^17^Department of Human Genetics, Radboud University Medical Center, Nijmegen, Netherlands; ^18^Department of Pathology, Radboud University Medical Center, Nijmegen, Netherlands

**Keywords:** predictive tumor markers, carcinoma, non-small cell lung, molecular targeted therapy, molecular pathology, precision medicine

## Abstract

The diagnostic landscape of non-small cell lung cancer (NSCLC) is changing rapidly with the availability of novel treatments. Despite high-level healthcare in the Netherlands, not all patients with NSCLC are tested with the currently relevant predictive tumor markers that are necessary for optimal decision-making for today's available targeted or immunotherapy. An expert workshop on the molecular diagnosis of NSCLC involving pulmonary oncologists, clinical chemists, pathologists, and clinical scientists in molecular pathology was held in the Netherlands on December 10, 2018. The aims of the workshop were to facilitate cross-disciplinary discussions regarding standards of practice, and address recent developments and associated challenges that impact future practice. This paper presents a summary of the discussions and consensus opinions of the workshop participants on the initial challenges of harmonization of the detection and clinical use of predictive markers of NSCLC. A key theme identified was the need for broader and active participation of all stakeholders involved in molecular diagnostic services for NSCLC, including healthcare professionals across all disciplines, the hospitals and clinics involved in service delivery, healthcare insurers, and industry groups involved in diagnostic and treatment innovations. Such collaboration is essential to integrate different technologies into molecular diagnostics practice, to increase nationwide patient access to novel technologies, and to ensure consensus-preferred biomarkers are tested.

## Introduction

Worldwide, an estimated 1.2 million–1.4 million deaths occur annually from non-small cell lung cancer (NSCLC) (1, 2). Most patients present with locally advanced or metastatic disease, for which the 1-year survival rate is around 20% (3, 4). In the era of precision medicine, NSCLC is no longer considered a single entity, but rather a heterogeneous disease comprising molecularly defined tumor subgroups that require individualized treatment (5, 6). The current article summarizes the opinions of 18 Dutch experts assessed during a workshop held on December 10, 2018, regarding the harmonization of approaches for optimal molecular diagnostics accessible for all patients with advanced-stage NSCLC.

To meet the need for standardization and harmonization initiatives for diagnostics and treatment, the expert advisory panel covered a range of disciplines, and included pulmonary oncologists, clinical chemists, pathologists, and clinical scientists in molecular pathology. The objectives of the workshop were to bring together different stakeholders and facilitate cross-disciplinary discussions for a consensus on current standards of practice for NSCLC diagnostics in the Netherlands; address relevant developments to provide a coherent perspective on future practice; identify challenges and gaps in the trajectory toward future practice; and disseminate the workshop findings.

## Scientific Background

The diagnostic landscape of NSCLC is changing rapidly with the availability of novel treatments. When activating mutations of the epidermal growth factor receptor (*EGFR*) gene or anaplastic lymphoma kinase (*ALK*) gene rearrangements are present, tyrosine kinase inhibitor treatment can significantly improve survival (7, 8). Currently in the Netherlands, 70–75% of stage IV non-squamous NSCLC patients are tested for *EGFR* mutations or *ALK* rearrangements (9). Additional predictive biomarkers are emerging, including those involving expression levels of programmed cell death-ligand 1 (*PD-L1*) (10) and genetic aberrations of ROS proto-oncogene 1 (*ROS1*), B-Raf proto-oncogene (*BRAF*), MET proto-oncogene (*MET*), RET proto-oncogene (*RET*), and erb-b2 receptor tyrosine kinase 2 (*ERBB2*; encoding *HER2*) (11, 12) and more recently of neurotrophic receptor tyrosine kinase 1 (*NTRK1*), *NTRK2, NTRK3*, and neuregulin 1 (*NRG1*) (13). Given that predictive biomarker validation and implementation in the clinic struggle to match scientific advancements and new drug approvals, continuous harmonization of the panel of biomarkers is needed, and testing for each patient's tumor prior to treatment initiation should be pursued.

Compared with platinum-containing chemotherapy, immuno-oncology (IO) agents that target tumors expressing programmed cell death-1 (PD-1) and PD-L1 improved overall survival in PD-L1-expressing advanced NSCLC (14). In the Netherlands, pembrolizumab has replaced cytotoxic chemotherapy as first-line treatment. Pembrolizumab and other IO agents are partnered with validated, but distinct PD-L1 immunohistochemical (IHC) companion and complementary diagnostic assays, which, despite similar analytical performance, may lead to interchangeability issues (15). This has led to ongoing efforts in PD-L1 testing harmonization to facilitate laboratory implementation and quality testing (16–18). Yet, just 2 years after the approval of pembrolizumab in patients with a PD-L1 tumor proportion score of 50% or greater, a phase 3 study of patients with untreated metastatic disease without *EGFR* mutations or *ALK* rearrangements/fusions reported that adding pembrolizumab to standard chemotherapy also improves overall survival compared with chemotherapy alone, regardless of PD-L1 status (19). The pace of these developments is evident and reveals a tension between validation and implementation of new biomarkers vs. the clinical utility and drug labels that necessitate the need for implementation. Other developments have added to this complexity:

- The IO drug durvalumab received European Medicines Agency approval for locally advanced, unresectable NSCLC following definitive chemoradiotherapy in adults whose tumors express PD-L1 on ≥1% of tumor cells (20).
- Tumor mutational burden as a predictor for IO response as yet lacks standardization and an understanding of its clinical utility and value in clinical practice (21).
- Liquid biopsy (a process for detecting molecular signatures originating from the tumor), including circulating tumor deoxyribonucleic acid (ctDNA) analysis, may cover gaps caused by sample quality/quantity issues associated with tissue biopsy, the longstanding gold standard for cancer diagnosis (22, 23).
  ◦ In 2016, the United States Food and Drug Administration granted approval for the first polymerase chain reaction (PCR)-based liquid biopsy assay (Cobas EGFR v2), which can be used as a companion diagnostic for *EGFR* mutations associated with NSCLC (24–26).
  ◦ There has been a shift from single-marker PCR-based testing for ctDNA to broad next-generation sequencing (NGS) panels capable of testing large numbers of predictive markers and different mutation classes in a single test (26, 27).
  ◦ Although ctDNA kits have become available for oncology research, guidelines for and routine clinical implementation of ctDNA testing continue to lag (22).
  ◦ Despite a joint review in March 2018 concluding that there is insufficient evidence of clinical validity and utility for most ctDNA assays in advanced cancer (28), more recent data are emerging to support the incorporation of ctDNA plasma NGS into routine NSCLC management (29, 30).

These and other advances raise questions regarding the optimal diagnostic pathway in non-squamous NSCLC cases being considered for personalized therapy. Given the broad range of test methodologies relevant for predictive markers and their application, an understanding and clinical guidance are needed of the different diagnostic tests for available and emerging biomarkers. These include tumor mutational burden and PD-L1, serum tumor markers, and ctDNA detected with digital PCR (dPCR) approaches or NGS, used either alone or as a complement to tissue diagnostics.

## Method

The discussions of the expert panel were complemented by a literature search and disseminated for peer review.

## Challenges That Impact Future Practice

### Challenge 1: A Predefined Panel of Diagnostic Markers Is Required

Following routine diagnosis of non-squamous NSCLC, preservation of sufficient tissue for predictive IHC and molecular testing is key (31–34). A predefined panel of markers is needed that can reliably be tested on relatively small tumor biopsies and with short turnaround time. There is also a need for testing of continuously emerging biomarkers. From a local perspective, uptake in molecular diagnostics is changing. For example, among 6,600 patients diagnosed with stage IV non-squamous NSCLC in the Netherlands, testing for *EGFR* mutations in tumor tissue increased from 24% of cases in 2013 to 44% in 2015 (9). Of the tested tumors that were found to be negative for mutations in *EGFR* and Kirsten rat sarcoma viral oncogene homolog (*KRAS*; *KRAS* mutations are considered to be mutually exclusive with *ALK* fusions), 50% in 2013 and 77% in 2015 underwent testing for *ALK* rearrangements/fusions. Gaps in testing may be related to both scarcity of tissue biopsy material, especially from stage IV disease, as well as unawareness of current testing possibilities. Currently, university laboratories are more likely than non-academic laboratories to use NGS (9, 35, 36).

The current guidelines in the Netherlands (37) are in accordance with international guidelines (11, 13, 38, 39) and recommend that molecular testing for *EGFR* mutations and *ALK* rearrangements/fusions should be performed in patients with metastatic (non-squamous) NSCLC for whom curative treatment is no longer available. In addition, the same 2015 guidelines strongly suggest to consider molecular testing for *KRAS, ROS1, RET, ERBB2*, and *BRAF* (37), comparable with the most recent international guidelines (13, 39). The consensus of the experts was that there is sufficient evidence to test not only for *EGFR* mutations and *ALK* rearrangements/fusions, but that all patients with advanced-stage non-squamous NSCLC should be tested for PD-L1 protein expression with IHC and for alterations associated with *BRAF, ERBB2, KRAS, MET, NRG1, NTRK1, NTRK2, NTRK3, RET*, and *ROS1* (Table 1). This allows for treatment in routine practice and provides access to ongoing trials and named-patient/early-access programs. There are further considerations in reaching this consensus:

- Testing is dependent on disease setting and treatment line, as well as methodology, since at the moment it is still possible to use techniques other than NGS and comply with guidelines.
- Certain drugs for targeted and immunotherapy appropriate for any given patient will not always be available at the initial location of care provided, and thus patients will need to be referred (requiring coordination of care between treating and referral centers).
- There is currently not enough clinical evidence to recommend routine testing for tumor mutational burden and microsatellite instability in NSCLC.

**Table 1:** Current and proposed molecular testing for NSCLC in the Netherlands.

	**Current guidelines**	**Consensus**
Recommended	ALK^*^, EGFR	ALK^*^, BRAF, EGFR, ERBB2, KRAS, MET, NRG1, NTRK1, NTRK2, NTRK3, PD-L1^**^, RET, ROS1
Strongly suggested	*BRAF, ERBB2, KRAS, RET, ROS1*	

* *Testing with IHC and mutation analysis*,

** *Testing with IHC*.

Except for PD-L1 and ALK, which require IHC for detection, the other markers should preferably be determined simultaneously using DNA and RNA-based NGS (supplemented only when necessary with *in situ* hybridization), both to conserve tissue biopsy material and decrease time-to-decision. As this technology evolves, molecular profiling will likely shift from its dominant application in late-stage non-squamous NSCLC to earlier disease stages where its use may maximize patient outcomes (40). Findings are awaited of the national, multicenter Lung Cancer Early Assessment (LEMA) trial in the Netherlands, which is enrolling 1,300 patients to investigate whether comprehensive molecular profiling across stage I–IV disease upon suspicion of NSCLC will improve diagnostic efficiency and patient benefit from personalized therapy (clinicaltrials.gov: NCT02894853; available from: https://clinicaltrials.gov/ct2/show/NCT02894853).

Since new therapies and biomarkers emerge quickly, regular updates to the panel of required biomarkers are needed, together with frequent modular updates of the multidisciplinary guidelines, including any biomarkers that are close to clinical introduction. Because official guidelines are based on evidence-based data only, the recommendations will be restricted to a limited set of predictive markers, comparable with other international guidelines. The major challenge for optimal therapy is also testing for those predictive markers for which specific drugs are not yet available in standard practice but are deemed to become available on short notice because of an already established beneficial toxicity and efficacy profile. Recent examples are *MET* or *NTRK* targeted therapy, at present only available through participation in company-sponsored clinical trials or by off-label or compassionate use. In many countries, off-label prescription of therapy is likely not reimbursed. Consequently, the legal and financial issues regarding the diagnostic testing are often not yet arranged. It is, however, important that laboratories offer the flexibility to physicians and their patients to choose between panels based on official guidelines in their (local) hospital and more expanded panels, including biomarkers that may enable access to clinical trials or non-registered drug–biomarker combinations.

The experts recommend the formation of a multidisciplinary committee, endorsed by national specialist medical societies, to provide annual guidance on biomarker panel content.

### Challenge 2: ctDNA Analysis Is Coming of Age, but Requires Standardization for Disease Profiling

Together with a shift from sequential, single-target molecular testing to concurrent, massively parallel testing using NGS, the development of liquid biopsy testing and ctDNA detection, in particular, offers a dynamic, comprehensive, and minimally invasive tumor-profiling approach (41). Analysis of ctDNA may be useful in cases where technical issues prevent tissue analysis or when patients are unable or unwilling to undergo biopsy (42). As a primary option, ctDNA may negate the need for tissue biopsy in patients with positive findings; subsequent tissue biopsy would maintain its utility in patients with negative findings, among whom high false-negative rates have been reported (30, 43, 44). Improved sensitivity of ctDNA testing is desirable, with positive findings associated with high tumor burden and negative findings more likely to have a reduced tumor load (45, 46).

Blood sampling for ctDNA testing is more easily performed than tissue biopsy, with potentially faster results compared with the longer turnaround times needed for tissue biopsies (30). Additional advantages of liquid biopsy include the potential for repeat sampling, and a more accurate representation of overall disease status than a single-site tissue biopsy (47). Health economic research is needed to confirm the cost–effectiveness of ctDNA testing and facilitate its clinical use (48). Finally, ctDNA analysis may identify markers associated with treatment response and resistance and, in early NSCLC, offer utility in identifying patients at high recurrence risk (40, 46). Disadvantages of ctDNA testing include limited sensitivity, restricted clinical utility, loss of a direct link between a mutation and a given lesion, and current technical issues regarding sensitive gene fusion detection in plasma. Where ctDNA itself is already a valid option for routine testing of actionable hotspot mutations, the technology (dPCR and/or NGS) can vary (49), and the use of broader panels vs. single markers in relation to the different applications in treatment-decision-making (26) needs to be further investigated. Further issues include lack of reimbursement, dependency on knowledge about the beneficial application of liquid biopsy, and interpretation of results.

### Challenge 3: Novel Diagnostic Tools Need to Be Made Available for All Patients

Access to new diagnostic techniques, such as liquid biopsy/ctDNA analysis, should be available for all patients. In practice, there are barriers accessing accurate and timely molecular diagnoses in oncology for reasons other than availability [e.g., capability to conduct testing, appropriate quality management systems (i.e., ISO 15189), geographic reach, guidelines] (36, 50). For *EGFR* mutation testing in NSCLC, external quality assessments have found room for quality improvements in diagnostic services based on a high level of diagnostic errors (24, 25, 51).

A regionally coordinated approach may assist in organizing complex molecular testing in accredited, staffed, and equipped laboratories (either academic or non-academic). This involves the development of capacity, logistics, and information sharing in centers equipped to deliver the latest diagnostic services normally associated with centralized laboratories. Successful implementation of a regionalized approach to molecular diagnostics also requires having an acceptable turnaround time of maximum 2 weeks. When questions justifying the reasoning, costs, cost–effectiveness, and reimbursement are adequately addressed, such an approach is more likely to be adopted.

On current evidence, it is difficult to definitively answer questions about molecular diagnostics. Limited data support a regionalized approach for patient care, but do not specifically shed light on molecular diagnostics. A recent US database analysis found that in NSCLC, treatment at a high-volume center was independently associated with improved overall survival (52). Given just 35% of cases were treated at such centers, regionalization of NSCLC management may improve patient outcomes. Although such NSCLC data are not available for the Netherlands, local data provide support for improved survival at high-volume centers for the systemic treatment of metastatic pancreatic cancer, but not for the surgical treatment of colorectal cancer (53, 54).

An understanding of cost and cost–effectiveness is even more problematic. Limited data suggest that techniques such as NGS are helpful in identifying mutations that can improve clinical decision-making at an affordable level (55). This leads into the issue of reimbursement, which is currently inadequate and consequently represents a barrier to the implementation of novel diagnostic innovations (56). Reimbursement in the Netherlands is further complicated by the Diagnosis Treatment Combination system, which restricts the healthcare provider in the choice of diagnostic tests during negotiations with the insurer. New diagnostic tests must meet the evidential requirements of individual hospital boards. Individual laboratories are thus required to negotiate increased internal funding to cover higher costs of molecular diagnostics. Normally, this occurs around historical cost data rather than expected costs of novel molecular tests. Thus, prospective health economic data are needed to support novel diagnostics, together with improved and unbiased reimbursement, greater transparency, and improved communication and collaboration between industry and stakeholders (57, 58).

### Challenge 4: Harmonization of Diagnostics Involves Several Different Steps

Despite consensus that molecular testing should be the standard of care in the management of patients with NSCLC (59), the biomarker landscape is rapidly changing. Awareness among centers of actionable drivers and their related biomarker-targeted treatments is an important first step toward harmonization, potentially leading to collaboration and broad access to diagnostic tools offering the greatest utility. This necessitates a consensus about which diagnostic tools and markers to adopt as standard care. Current issues that remain unresolved include the lack of harmonization in preanalytical conditions, varying performance, lack of comparison between diagnostic technologies, and the absence of a standardized approach in applying the different techniques (60).

Achieving a consensus over the minimal requirements and performance of any given diagnostic approach requires multidisciplinary collaboration between treating physicians, pathologists, clinical chemists, molecular laboratories, and other stakeholders (61). Healthcare institutions and payers need to be involved in any conversation. Questions for the former might seek to understand any requirements for personnel, specialized equipment, and administrative support. Ultimately, the aim of harmonization is to ensure a robust and comprehensive final diagnosis through which to direct treatment and achieve optimal outcomes. Therefore, the healthcare service provider requires not only the physical infrastructure, but also the communication networks to facilitate effective collaboration among healthcare professionals involved in providing the comprehensive diagnosis (62). This may require ongoing education to meet the needs of all stakeholders.

Given the continuous flow of upcoming biomarkers and novel technologies, a further step toward a validated, harmonized diagnostic approach is to extend the conversation to the pharmaceutical and diagnostics industry, which may help facilitate communication between medical specialists and healthcare institutions/payers. Harmonization is a long-term process that is ideally evidence-based yet also innovative, facilitates close interactions between the laboratory and clinician, and is a shared responsibility for all stakeholders (63). Industry involvement could help to establish pilot projects between healthcare institutions and insurers, and provide financial support through which to assess proposed diagnostic approaches for routine use, with a view to better understanding issues such as indications, tissue requirements, turnaround times, reliability, pitfalls, and costs (59, 61, 64, 65).

## Conclusions

The overarching challenge in NSCLC management is to implement the science behind predictive biomarkers into routine clinical practice where the aim is to translate diagnostic findings into an optimal, individualized treatment pathway. This requires collaboration between healthcare professionals involved in the diagnosis and treatment of NSCLC (medical specialists) (62), other stakeholders at the point of care (healthcare institutions), reimbursement (payers), and innovation (industry). Whereas NSCLC diagnosis has proceeded gradually in the molecular era, with biomarker testing often varying considerably between institutions (9, 66), it is important to align diagnostic approaches with clinical needs and to harmonize between laboratories. Recently, several projects have been initiated in the Netherlands to address the discussed issues. These projects aim at harmonization and evidence-based introduction of state-of-the-art biomarkers in diagnostic laboratories in the Netherlands. Examples of such projects are PATH (Predictive Analysis for Therapy; available from: https://www.netwerk-PATH.nl) and COIN (ctDNA on the road to implementation in the Netherlands; available from: www.cfDNA.nl). The coming years will show the value of such national initiatives in implementing new biomarkers. For the moment, it is recommended that molecular testing for metastatic NSCLC analyses is performed on tumor tissue or cytological material, and that the use of ctDNA analyses is reserved for those patients who cannot undergo tumor sampling.

The consensus of the experts was that all patients with advanced-stage non-squamous NSCLC should be tested for *EGFR* mutations and *ALK* rearrangements/fusions, PD-L1 protein expression with IHC, and alterations associated with *BRAF, ERBB2, KRAS, MET, NRG1, NTRK1, NTRK2, NTRK3, RET*, and *ROS1*.

Continuous adaptation of guidelines is needed to keep pace with scientific progress. The present discussion document identifies the main challenges with developing and implementing a harmonized approach to molecular diagnostics in NSCLC treatment. It is challenging to establish a role for novel technologies, such as ctDNA testing, and to make these routinely available for all patients. This requires awareness among stakeholders and necessitates multipartite collaboration to obtain a comprehensive understanding of a fully implemented diagnostic network that fulfills the clinical needs for routine care.

## Author Contributions

DB, TH, BB, WD, NH, JH, MPGL, KM, MO, VS, ES, E-JS, RS, JT, SW, LV, and MJLL discussed challenges at the advisory board. All authors further discussed content per email, contributed to writing/revising the manuscript, and provided final approval of the manuscript.

### Conflict of Interest

All authors received reimbursement for attending the advisory board meeting from Roche. DB reports fees from Roche Diagnostics, ZonMw, Merck, Health~Holland, and AstraZeneca; grants from Roche Pharma, MSD, Novartis, and Pfizer; and non-financial support from Biocartis. TH, BB, JH, MPGL, MO, VS, MH, and RS have nothing further to disclose. WD reports fees from Amgen, Bayer, Bristol-Myers Squibb, Novartis, and Roche; and grants from AbbVie, AstraZeneca, Bristol-Myers Squibb, Novartis, and Roche. NH reports grants from Roche and Pfizer. KM reports fees from Pfizer, Bristol-Myers Squibb, Roche, MSD, AbbVie, AstraZeneca, and Diaceutics; and grants from AstraZeneca, MSD, and Roche. ES reports fees from Roche, Biocartis, Bristol-Myers Squibb, Astra Zeneca, Novartis, Bio-Rad, Pfizer, Bayer, Illumina, Janssen-Cilag, Diaceutics, and Agena Bioscience; grants from Biocartis, Roche, CC Diagnostics, Boehringer Ingelheim, Bristol-Myers Squibb, Pfizer, Bio-Rad, Qiagen, Promega, and Agena Bioscience. ES reports fees from Roche; grants from AstraZeneca, Bristol-Myers Squibb, MSD, and Novartis; non-financial support from AbbVie; and other from Bristol-Myers Squibb, MSD, AbbVie, Pfizer, Roche, and Bayer. JT reports personal fees from Roche, MSD, and AbbVie; and grants from Bristol-Myers Squibb. SW reports grants and personal fees from Roche, Pfizer, Bristol-Myers Squibb, MSD, AstraZeneca, and NextCure. LV is a current employee of Roche. MJLL reports personal fees from Roche; grants from AstraZeneca and Bristol-Myers Squibb; non-financial support from Illumina; and other from Bayer, Janssen Pharmaceutica, Merck, and Nimagen.
